# Supplementary material for: Development of a Real-Time Quantitative PCR Assay for the Specific Detection of Bacillus velezensis and Its Application in the Study of Colonization Ability
Source: Microorganisms. 2022 Jun 14;10(6):1216. doi: 10.3390/microorganisms10061216 (PMC9230654; doi:10.3390/microorganisms10061216)
Supplement: Supplementary file 1 [file microorganisms-10-01216-s001.zip › microorganisms-1746244-supplementary.pdf]

Table S1 Primers designed in this study

Targeted strain	Targeted genes	Primer name	Primer sequence (5′ to 3′)	Product size	Tm amplicon °C
Bacillus velezensis ZF2	*galE*	GA-F1	CTTCCCAGCCTAATTCAGCC	160 bp	61.1
		GA-R1	GAACACAACAGGCGCGG		62.5
Bacillus velezensis ZF2	*galE*	GA-F2	CACACCGTGTGCCTGCA	220 bp	62.8
		GA-R2	GCATTGGAGCGTGTGAAAGATA		62.4
Bacillus velezensis ZF2	*gyrB*	GY-F1	AGGAGAAGATGGGCCGC	180 bp	61.3
		GY-R1	GCTCGTACGCCTGATAGTGG		60.8
Bacillus velezensis ZF2	*gyrB*	GY-F2	TTAAGCGGGGATGATGTGAG	260 bp	61.0
		GY-R2	CAAGCGCACTTTTGCGG		62.7
Bacillus velezensis ZF2	*metC*	ME-F1	TATCAACGAAGTGGCGCG	210 bp	61.9
		ME-R1	GTCCGCTCAGGAATTTTGTTG		61.9
Bacillus velezensis ZF2	*pdhA*	PD-F1	GGAATTAATGAGAAGAATGGTGTATATC	230 bp	58.8
		PD-R1	CTGATTACCTTTAAAATGGCCTC		58.3
Bacillus velezensis ZF2	*pdhA*	PD-F2	TTGCCGTCGGTATTACAGGT	380 bp	60.4
		PD-R2	GGCTCTTCAAACATATTCTCAATCA		60.8
Bacillus velezensis ZF2	*pdhA*	PD-F3	CAGCGCGCAATTAACGGT	280 bp	63.2
		PD-R3	ATTCTCAATCAGCTCTGTTACTTTCG		61.4
Bacillus velezensis ZF2	*pgk*	PG-F1	GGCTATGAAGTCGGTAAATCTCTT	160 bp	59.2
		PG-R1	GATTTGAACGTTTGCATCG		57.7
Bacillus velezensis ZF2	*fliC*	FL-F1	CCAGCAGCTTTTGTGACTTC	200 bp	58.6
		FL-R1	GCAAAACCTTACGTTCCAAAT		58.2
Bacillus velezensis ZF2	*fliC*	FL-F2	GCAACAACTTTACCTTTATCATCA	179 bp	57.5
		FL-R2	TCAAAATACACTTACAGCAACT		52.1
Bacillus velezensis	*fliC*	FL-F3	TCCAGCAGCTTTTGTGACTTC	102 bp	60.6

ZF2		FL-R3	TCTGGTGATCAAAATACACTTAC		53.7
Bacillus velezensis ZF2	*fliC*	FL-F4	CAGCCCAAGTAGCAGTGC	161 bp	58.1
		FL-R4	GCAAAACCTTACGTTCCAAAT		58.2
Bacillus velezensis ZF2	*fliC*	FL-F5	TTTTCAGATGCAACAACTTTACC	103 bp	58.4
		FL-R5	GCACTGCTACTTGGGCTG		58.1
Bacillus velezensis ZF2	D3N19_RS01160	1-F	GTTGTCACATCGTACTCATAATCAA	202 bp	58.1
		1-R	CTTAACCAAACAAACAAGCATTC		57.9
Bacillus velezensis ZF2	D3N19_RS01185	2-F	GTCGGTCAATCTTGTTTCACA	148 bp	58.6
		2-R	CAATGTTTTTTCACGCCTTT		57.8
Bacillus velezensis ZF2	D3N19_RS02665	3-F	CACAATAGAGCTGTATTAAGGGG	200 bp	57.1
		3-R	CCAGGAAAGAAGGATGAAATT		57.3
Bacillus velezensis ZF2	D3N19_RS04495	4-F	GCATTTGCTCTTTTTATGTTTCA	214 bp	58.5
		4-R	CTAGTATAACAGCTCATAAAAAGGC		55.3
Bacillus velezensis ZF2	D3N19_RS06040	5-F	TTATTACCCTCTTTTTCTGCAGTT	159 bp	57.7
		5-R	GGCTTCTTTTATAATTTCAATTTCA		57.3
Bacillus velezensis ZF2	D3N19_RS06715	6-F	CATAATGAAATTGAAAGGAAAAAGA	199 bp	57.9
		6-R	GAAGTATCTGTACAGGAGAAATAGAGA		55.9
Bacillus velezensis ZF2	D3N19_RS09280	7-F	GTGCGGAAACTGGGTAACAT	160 bp	59.6
		7-R	CAATCGACATTCCAAGCAG		57.8
Bacillus velezensis ZF2	D3N19_RS09770	8-F	CGATCCGTATCTTCAATTGTTC	137 bp	58.6
		8-R	ATGAAAAAAACAGAGCAAGACAA		58.1
Bacillus velezensis ZF2	D3N19_RS12345	9-F	GTTTTCAAGTGTGGCATAGGTAA	195 bp	58.2
		9-R	CTGTAAACTGCTTCCGG		52.5
Bacillus velezensis ZF2	D3N19_RS12385	10-F	CGGGCGTAAAAAAGGAGA	186 bp	58.8
		10-R	GAAGGGGTTAAATATCAATAAAAA		55.1
Bacillus	**D3N19_RS13500**	**11-F**	**CTAATTTTTCCTATTTCTTTAACGC**	**193 bp**	**56.4**

***velezensis* ZF2**		**11-R**	**ATTTATTTAAATTCACTTACATCAGT**		**52.2**
Bacillus velezensis ZF2	D3N19_RS14285	12-F	GGCGGCAGACAGTACAATATC	251 bp	59.6
		12-R	CAAACAACTGATAAAAAGACATTTC		56.3
